# Public Health, One Health, and Planetary Health: what is next?

**DOI:** 10.1093/eurpub/ckae149

**Published:** 2024-10-08

**Authors:** Francisco Olea-Popelka, Nicole Redvers, Saverio Stranges

**Affiliations:** Department of Epidemiology and Biostatistics, Schulich School of Medicine & Dentistry, Western University, London, Ontario, Canada; Department of Epidemiology and Biostatistics, Schulich School of Medicine & Dentistry, Western University, London, Ontario, Canada; Department of Epidemiology and Biostatistics, Schulich School of Medicine & Dentistry, Western University, London, Ontario, Canada; Department of Family Medicine, Western University, London, Ontario, Canada; Department of Medicine, Western University, London, Ontario, Canada; Department of Clinical Medicine and Surgery, University of Naples Federico II, Naples, Italy

The still predominant siloed, vertical structure of academia, health care systems, funding institutions/mechanisms, and public health organizations around the world pose an important challenge to tackle complex societal and health challenges for people, animals, and ecosystems. Understanding and acknowledging the delicate interdependence between ecosystem, human, and animal health is needed to design and implement comprehensive and holistic health strategies, beyond just human health. Infectious diseases with a zoonotic component have caused widespread human suffering in recent decades, with increased interactions between human and animal populations making people ever more vulnerable to new infections, given the rapidly and constantly changing global ecosystem. Additionally, socio-cultural, political, and economic factors impact the ability of systems to better prevent, detect, and respond to public health challenges at the human, animal, and environmental interface. This complex landscape applies to non-communicable diseases as well, requiring multisectoral approaches well beyond the traditional, narrow biomedical model. Hence, a wholesale shift is needed in how we approach public health. Instead of equating public health only with human health, we need to recognize what it truly is: the inter-related health of the world’s people, animals, and the environments we all share.

The complete interdependence between human, animal, and ecosystem health has been long recognized within Indigenous communities; however, the emergence and rapid expansion of the fields and practice of both One Health (OH) and Planetary Health (PLH) are recent developments in the right direction [1]. While the OH approach has been advocated for mostly in the context of addressing global threats related to zoonotic diseases and antimicrobial resistance, this approach is also relevant for several major public health challenges including pollution management, the environmental/agricultural component of food safety, food security, and nutrition. For example, the OH approach may lead to ecologically sustainable dietary patterns impacting the prevention and management of chronic conditions, such as cardiovascular disease.

The OH/PLH approaches have experienced considerable growth and expansion in academia, and within governmental and non-governmental organizations (NGOs)—with greater traction occurring in the past decade [1]. While OH and PLH are highly complementary approaches based on transdisciplinary, multisectoral, and system-based approaches to health, challenges remain when translating ideas into policy and practice. “Overall, One Health and Planetary Health provide an opportunity to build a stronger research community to collectively address pressing public and global health issues in a truly integrated way” [1].

In March 2023, the Quadripartite organizations: the Food and Agriculture Organization of the United Nations (FAO), United Nations Environment Programme (UNEP), World Health Organization (WHO), and World Organisation for Animal Health (WOAH), issued an unprecedented call for enhanced global action to use the OH approach to “achieve together what no one sector can achieve alone” [2], emphasizing the need to translate the OH approach into policy action. Additionally, the Quadripartite institutions, in December 2023, published the One Health Joint Plan of Action [3] with recommendations to implement OH approaches at national levels. Similar movements are occurring within the PLH space. For example, a National Planetary Health Action Plan (NPHAP) is being developed in Malaysia “to mainstream planetary health in all national policies and plans through a holistic and whole-of-nation approach” [4]. Having endorsements from national and international organizations are important; however, there are still elements lacking when considering the implementation of OH/PLH to ensure human, animal, and ecosystem health.

***What is next?*** Local community leadership and involvement is needed to build upon progress to date at the global level. To tackle complex public health challenges, a “bottom-up” approach is needed that complements global and national efforts. An emphasis on local, practical, and feasible solutions are also needed to address complex problems, while engaging local stakeholders and affected communities. A key aspect, however, of implementing OH and PLH approaches into public health strategies is to account for the socio-cultural, religious, and economic factors among local and rural communities. This is especially important when working with those most marginalized, such as Indigenous and rural communities, who are often already closely and directly attached to having strong connections with the ecosystem they inhabit.

Scientific, biomedical, and health knowledge is necessary, but not sufficient alone. Successful public health interventions that work at the human-animal-ecosystem interface require the broad and committed collaboration of members from all levels of society. A coordinated, multisectoral approach that involves animal health and public health authorities, health practitioners, physicians, veterinarians, environmental workers, politicians, researchers, experts in social, cultural, and communication issues, as well as economists, farming and agricultural groups, and local communities is necessary. Importantly, bold and courageous political leadership is essential to co-lead while securing public support for health policy decisions and implementation [5]. It is key to develop a OH/PLH “business case” (e.g. cost-benefit analysis), with governments enabling, facilitating, and supporting implementation processes both financially and within appropriate legal frameworks. This will ensure the recognition for not only the importance of economic benefits derived from reducing a specific health issue, but also for assessing the broader public health and societal benefits and impacts.

While OH and PLH offer a rational systems approach for safeguarding health in an interconnected world, to secure its benefits, public health must do what humans, animals, and plants have always done—***evolve***!
